# Nanosilver–Silica Composite: Prolonged Antibacterial Effects and Bacterial Interaction Mechanisms for Wound Dressings

**DOI:** 10.3390/nano7090261

**Published:** 2017-09-06

**Authors:** Dina A. Mosselhy, Henrika Granbohm, Ulla Hynönen, Yanling Ge, Airi Palva, Katrina Nordström, Simo-Pekka Hannula

**Affiliations:** 1 Department of Chemistry and Materials Science, School of Chemical Engineering, Aalto University, 02150 Espoo, Finland; henrika.granbohm@aalto.fi (H.G.); yanling.ge@aalto.fi (Y.G.); simo-pekka.hannula@aalto.fi (S.-P.H.); 2 Microbiological Unit, Fish Diseases Department, Animal Health Research Institute, Dokki, Giza 12618, Egypt; 3 Department of Veterinary Biosciences, Division of Veterinary Microbiology and Epidemiology, University of Helsinki, P.O. Box 66, 00014 Helsinki, Finland; ulla.hynonen@helsinki.fi (U.H.); airi.palva@helsinki.fi (A.P.); 4 Department of Bioproducts and Biosystems, School of Chemical Engineering, Aalto University, 02150 Espoo, Finland; katrina.nordstrom@aalto.fi

**Keywords:** silver nanoparticles, silica, composite, prolonged silver leaching, antibacterial effects, mechanisms of action, wound dressings

## Abstract

Infected superficial wounds were traditionally controlled by topical antibiotics until the emergence of antibiotic-resistant bacteria. Silver (Ag) is a kernel for alternative antibacterial agents to fight this resistance quandary. The present study demonstrates a method for immobilizing small-sized (~5 nm) silver nanoparticles on silica matrix to form a nanosilver–silica (Ag–SiO_2_) composite and shows the prolonged antibacterial effects of the composite in vitro. The composite exhibited a rapid initial Ag release after 24 h and a slower leaching after 48 and 72 h and was effective against both methicillin-resistant *Staphylococcus aureus* (MRSA) and *Escherichia coli* (*E*. *coli*). Ultraviolet (UV)-irradiation was superior to filter-sterilization in retaining the antibacterial effects of the composite, through the higher remaining Ag concentration. A gauze, impregnated with the Ag–SiO_2_ composite, showed higher antibacterial effects against MRSA and *E*. *coli* than a commercial Ag-containing dressing, indicating a potential for the management and infection control of superficial wounds. Transmission and scanning transmission electron microscope analyses of the composite-treated MRSA revealed an interaction of the released silver ions with the bacterial cytoplasmic constituents, causing ultimately the loss of bacterial membranes. The present results indicate that the Ag–SiO_2_ composite, with prolonged antibacterial effects, is a promising candidate for wound dressing applications.

## 1. Introduction

The skin is the largest body organ, forming a protective barrier against harmful bacteria. Skin damage allows for bacterial penetration, enabling local wound infections or systemic septicemia [1]. Healing of acute wounds is an orderly and timely regenerative process. Therefore, the management of acute wounds essentially means preventing complications, such as wound infections, which can halt the regeneration process of tissues and convert acute wounds to chronic wounds [2]. As classified in terms of the depth of the skin injury, superficial wounds comprise injuries of the epidermis and papillary dermis only and are healed within ten days, provided that infections have been prevented [1]. Wound dressings vary according to the type of wound [3] and play a vital part in wound healing [4,5,6,7] by acting as physical barriers and by preventing wound contamination and infection [1]. Topical antibiotics are administered for the initial treatment of infected superficial wounds [8]. However, the unbridled use of antibiotics has resulted in the emergence of bacterial antibiotic resistance [9,10,11]. There is a growing concern that these highly resistant bacterial populations may be opening up an era of non-treatable infections [12]. Most notably, the increase of serious infections caused by MRSA is alarming, conventionally in hospital environments and wound care [13,14], and may lead to the death of patients [4]. In addition, MRSA is now a predominant pathogen in the community reservoir as well [15,16], also causing fatal infections [17]. This will increase the administration of vancomycin [15], a glycopeptide antibiotic that is considered as an ultimate arsenal for treating MRSA [18], which may provoke the tenacity of antibiotic-resistant Gram-positive bacteria [15]. To further complicate the resistance quandary, vancomycin resistance has already been identified in MRSA [19]. Accordingly, the emergence of antibiotic-resistant bacteria calls for the rapid formulation of new therapeutic modalities that are less likely to promote the development of bacterial resistance [10,14,20]. 

Silver (Ag) has received resurgent interest for use in medicine, particularly in wound management [4,21,22,23]. Notably, Ag in wound dressings has shown promising antibacterial effects [24] and it has been shown that silver nanoparticles (Ag NPs) are highly antibacterial agents [25,26,27]. The weaker tendency of Ag to elicit bacterial resistance is due to the complex interference of Ag NPs and released silver ions (Ag^+^) with bacterial cells [24]. For instance, the interaction of Ag NPs with the bacterial cell membranes leads to the formation of “pits” and damage to the membranes, which increase the permeability of membranes, resulting in bacterial death [28]. Moreover, Ag NPs can form free radicals that cause damage to the membranes, leading to an antibacterial effect [29]. Furthermore, Ag^+^ can interact with phosphorus moieties in DNA, hindering bacterial replication, as well as interfere with sulfur-containing proteins in the bacterial cell walls and thiol groups of bacterial enzymes, resulting in their damage and inactivation [30]. Consequently, Ag-based dressings are generally preferred in the topical management of wound infections, diabetic wounds [31,32], and particularly in the prophylaxis and control of infections caused by antibiotic-resistant bacteria [4,21,33]. On the other hand, Ag NPs are susceptible to aggregation, which results in loss of their antibacterial properties [34,35]. 

Silica (SiO_2_) particles can be efficiently utilized as a stabilizing matrix for preventing the aggregation of Ag NPs [7,27,36,37,38]. Moreover, SiO_2_ particles have high chemical and thermal stabilities, are inert and biocompatible, which propose them as an excellent system to deliver antibacterial agents [11]. Immobilization of Ag NPs can also provide prolonged antibacterial effects, as Ag^+^ have been shown to exhibit sustained release from the immobilized Ag NPs on substrates [39]. One approach to immobilize Ag NPs is by utilizing the core-shell systems. The main challenges in this approach are the aggregation of the Ag cores when decreasing the thickness of SiO_2_ shells and the slow dissolution rate of the Ag cores when increasing the shell thickness [40]. Such characteristics can prevent the full utilization of Ag NPs in the core-shell systems. Therefore, in this study, to maximize the benefits of immobilization and the prolonged release of Ag to safeguard the antibacterial effects in wound dressing applications, we have developed a composite by immobilizing Ag NPs on SiO_2_ matrix. 

Previous studies have described broad-spectrum antibacterial effects for Ag–SiO_2_ composites, with more efficacy against Gram-negative bacteria [41,42,43]. The present study, in turn, aims to investigate the prolonged antibacterial performance of the composite against both Gram-positive and Gram-negative bacteria (MRSA and *E*. *coli*, respectively). As MRSA and *E*. *coli* are common wound pathogens [13,44], therefore, the examination of their sensitivity to the composite is of particular interest considering the proposed wound dressing applications. Whilst the administration of Ag-containing dressings is increasing, debate continues concerning their efficacy [4]. At present, there is only little published data on the antibacterial efficacy of the dressings that have recently reached the market. It has even been demonstrated that there is no direct relation between the Ag content, Ag release and the antibacterial effects of the Ag-containing dressings, and that a high release rate of Ag from the dressings is not a guarantee for their antibacterial efficacy [45]. Therefore, we have also compared the antibacterial effects of a currently available commercial Ag-containing dressing (CSD) with the Ag–SiO_2_ composite-impregnated gauze (Ag–SiO_2_-G) in vitro. The specific objectives of this study are (i) the preparation and characterization of a Ag–SiO_2_ composite; (ii) the determination of the leaching profile and the prolonged antibacterial effects of the composite against MRSA and *E*. *coli*, in comparison with a CSD, with the aim of acute wound management and infection control; and (iii) the identification of the antibacterial mechanisms of the composite.

## 2. Results and Discussion

### 2.1. Characterization of Ag–SiO_2_ Composite and SiO_2_ Particles

The prepared Ag–SiO_2_ composite and SiO_2_ particles were characterized utilizing a range of instrumental techniques, such as X-ray diffraction (XRD), scanning electron microscope (SEM), transmission electron microscope (TEM), high-resolution TEM (HRTEM), energy dispersive X-ray spectroscopy (EDX) of the scanning transmission electron microscope (STEM), and Zetasizer. The XRD patterns of the Ag–SiO_2_ composite and SiO_2_ particles are displayed in Figure S1. The humps around 25° (2*θ*) in both patterns are attributed to the amorphous structure of the SiO_2_. The XRD pattern of the composite does not reveal diffraction peaks for the crystalline Ag. The absence of diffraction peaks for the Ag NPs can be attributed to the small size of the Ag NPs obtained at the low heating temperature (300 °C) of the composite. This is consistent with a previous research [46] that has also reported the absence of diffraction peaks for the immobilized Ag on SiO_2_ at 400 °C heat treatment in air and detected Ag diffraction peaks only when the mean size of Ag NPs increased with the increase of the heating temperature. This relationship between the absence of diffraction peaks and the small size of Ag NPs, 7 to 9 nm [47], and 2 to 3 nm [48], has further been reported in the literature.

**Figure 1:**
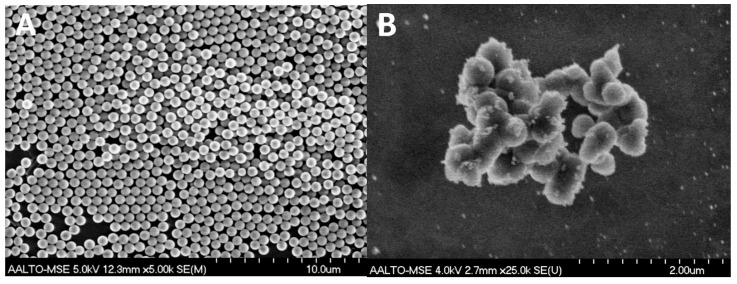
SEM images showing (**A**) the spherical pristine SiO_2_ particles; and (**B**) the raspberry-like Ag–SiO_2_ composite with surface-immobilized Ag NPs.

**Figure 2:**
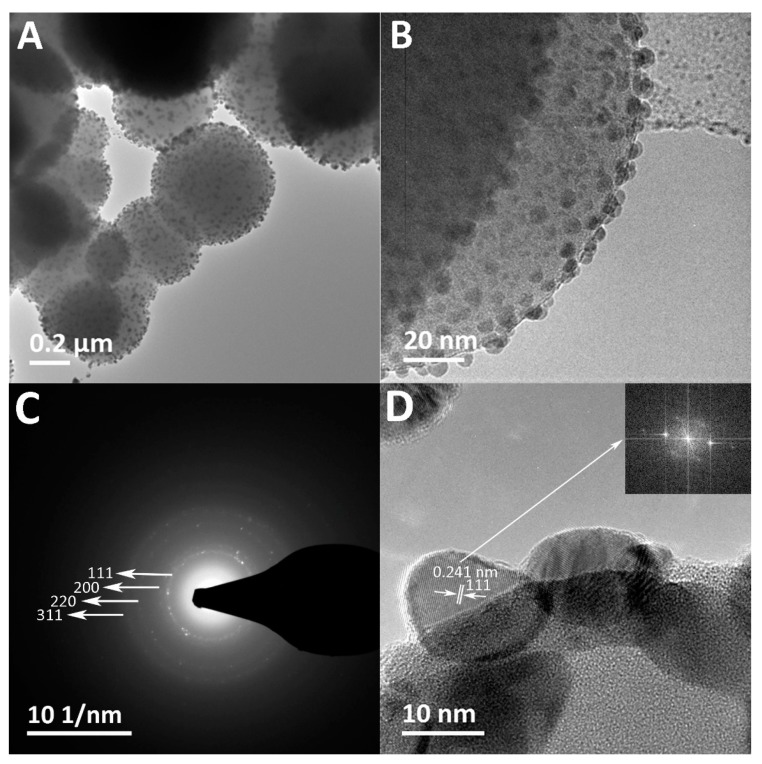

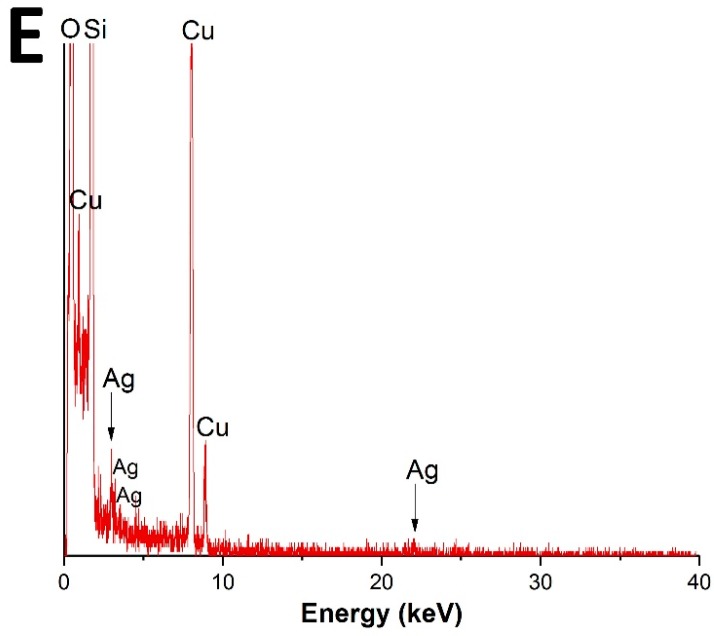
(**A**,**B**) TEM images showing spherical Ag NPs immobilized throughout the SiO_2_ matrix in the raspberry-like composite at different magnifications; (**C**) The selected-area electron diffraction (SAED) ring pattern of the crystalline Ag NPs of the composite; and (**D**) the high-resolution TEM (HRTEM) image of the labeled surface-immobilized Ag NP showing the lattice fringes (*d*-spacing) and the corresponding fast Fourier transform (FFT) pattern (inset); (**E**) The energy dispersive X-ray spectroscopy (EDX) elemental analysis of the Ag–SiO_2_ composite.

The SEM images show the surface morphology of the spherical pristine SiO_2_ particles (Figure 1A) with median and average sizes of 673 nm and 674 ± 22 nm, respectively (Table 1), and the raspberry-like Ag–SiO_2_ composite with the surface-immobilized Ag NPs exposed (Figure 1B). The TEM images (Figure 2A,B) reveal the spherical, relatively dark Ag NPs immobilized all over the SiO_2_ matrix forming a raspberry-like composite. The median and average sizes of the Ag NPs of the composite are 5 nm and 5 ± 2 nm, respectively, with a size distribution ranging from 2 to 20 nm (Table 1 and Figure S2). This small size of Ag NPs has an implication considering the size-dependent antibacterial effects of Ag NPs: smaller Ag NPs, preferably in the range of 1 to 10 nm, have shown better antibacterial effects than larger ones [49,50]. Furthermore, the TEM images display the Ag NPs with a uniform distribution throughout the SiO_2_ matrix without aggregation. This uniform distribution is favorable, as aggregation reduces the active surfaces of Ag NPs, and thus results in loss of their antibacterial effects [34,42].

The selected-area electron diffraction (SAED) pattern of the composite (Figure 2C) shows the ring pattern with the *d* values calculated, corresponding to plane spacing of the {111}, {200}, {220}, and {311} planes of the face-centered cubic (fcc) crystal structure of Ag reported in the international centre for diffraction data (ICDD, reference code: 04-016-6676). The HRTEM images of the composite demonstrate (i) single-crystal Ag NPs as indicated by the one-directional lattice fringes in Figure 2D showing *d*-spacing of 0.241 nm, which matches the {111} plane spacing of the fcc Ag crystal; and (ii) twinned and multi-grain Ag NPs (Figure S3). The multi-grain Ag NPs may be attributed to the growth of the small single Ag crystals into larger Ag NPs [51]. Together these results provide important information on the crystalline structure of Ag NPs of the composite that has not been revealed by XRD. The EDX results (Figure 2E) show peaks of Ag, Si, and O, which further confirm the presence of Ag within the SiO_2_ matrix. The detected peaks of copper (Cu) are originating from the copper grid. The zeta potential values of the Ag–SiO_2_ composite and SiO_2_ particles are −68.3 ± 1 mV and −66.9 ± 0.7 mV, respectively, indicating the negative surface charge and the electrostatic stability of the prepared materials.

### 2.2. Ag Leaching Profile

**Figure 3:**
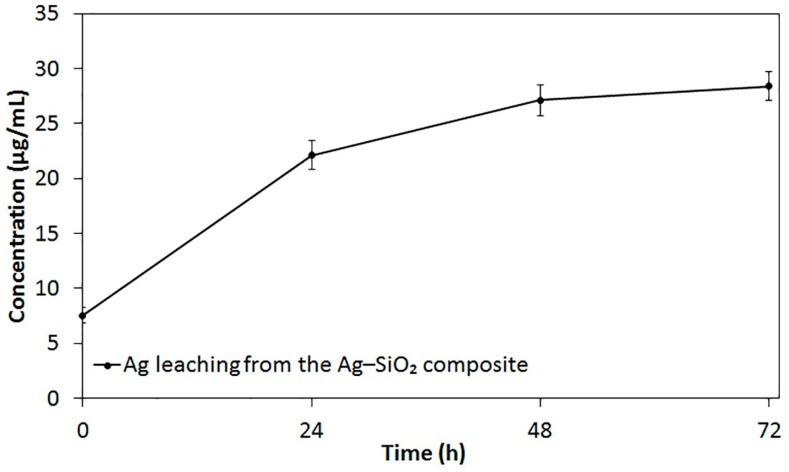
In vitro leaching profile of Ag from the filtered aqueous suspensions of the Ag–SiO_2_ composite (1 mg/mL), shaken at regular time intervals, shown as the average values of triplicate measurements. The bars represent the standard errors of the averages.

Inductively coupled plasma-optical emission spectrometer (ICP-OES) was utilized to identify the prolonged Ag release from the Ag–SiO_2_ composite. The total concentration of Ag in the non-filtered stock of Ag–SiO_2_ composite (1 mg/mL) is 57.8 ± 10.4 μg/mL (100%). The in vitro leaching profile of Ag from the filtered Ag–SiO_2_ composite as the function of time is shown in Figure 3. At the start of the experiment (0 h), the filtration of the stock suspensions had resulted in 7.5 ± 1.2 μg/mL Ag concentration, which represents ~13% of the stock Ag concentration. After 24 h, Ag was quickly leached from the composite with a concentration of 22.1 ± 2.3 μg/mL (~38.2%). Then, a slower sustained leaching of Ag was detected, as the concentrations of 27.1 ± 2.4 μg/mL (~46.9%) and 28.4 ± 2.2 μg/mL (~49.1%) were detected after 48 and 72 h, respectively. A possible explanation for the subsequent slower sustained release of Ag is the depletion of the immobilized Ag NPs from the surface of SiO_2_ particles.

Overall, the present results have three important implications. First, the initial quick leaching of Ag is desirable, as a rapid antibacterial action is a property of an ideal wound dressing [52]. Secondly, the sustained leaching of Ag allows for a prolonged antibacterial action of Ag. Thirdly, the remaining Ag concentration of the embedded Ag NPs throughout the SiO_2_ matrix should be interpreted with some caution, as if sub-lethal concentrations of Ag are released, Ag-resistance might evolve [22,24]. Ag-resistance genes have previously been documented in a plasmid of a *Salmonella* strain isolated from a hospital burn unit [53], and homologs of these genes have also been identified in *E*. *coli* chromosomes [54]. While the incidence of Ag resistance remains rare, clinicians and scientists should, however, be aware of the Ag concentrations needed to be administered for achieving the desired antibacterial effects of Ag, but simultaneously strive to avoid the emergence of resistance. It has been recommended that prolonged use of Ag-dressings should be avoided if wounds show no response to Ag after 3 to 5 times of changing dressings within 10 to 15 days [32].

### 2.3. Antibacterial Effects of Ag–SiO_2_ Composite and Dressings

**Figure 4:**
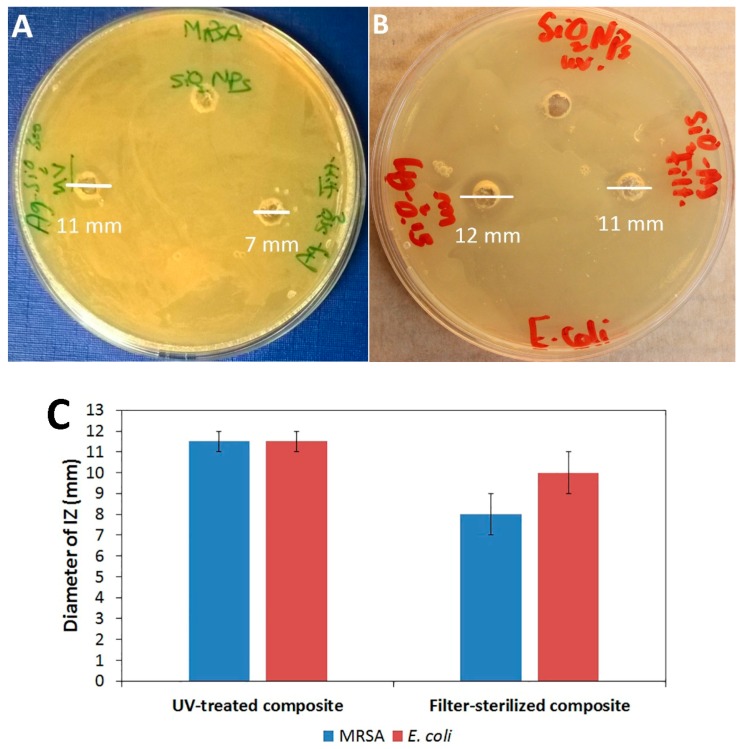
Antibacterial effects of UV-treated and filter-sterilized Ag–SiO_2_ composites detected by the diameters of inhibition zones (IZs) on plates with (**A**) MRSA and (**B**) *E*. *coli*. (**C**) The diameters of IZs. The averages and standard errors of two independent agar diffusion assays are shown.

The susceptibility of MRSA and *E*. *coli* to the Ag–SiO_2_ and SiO_2_ powders was tested in the first set of agar diffusion assays. No inhibition zones (IZs) are detected on plates of MRSA and *E*. *coli* with SiO_2_ particles (Figure 4A,B, respectively), which demonstrates that the SiO_2_ particles have no role in the antibacterial effects of the composite. In contrast, the Ag–SiO_2_ composite produces IZs of both MRSA and *E*. *coli*. The antibacterial effects of the Ag–SiO_2_ composite are most likely contributed to the small size (5 nm) of the Ag NPs. These small sized-Ag NPs possess large surface areas, enabling them to have large contact areas with the bacterial cells [26,50,55] and to release high amounts of Ag^+^ [27,56]. Moreover, the aerobic environments of the antibacterial tests allow for the partial surface oxidation of the Ag NPs. Partially oxidized Ag NPs possessing high levels of Ag^+^ may facilitate the antibacterial effects, as previously reported by Lok et al. [34]. The present findings are consistent with those of Agnihotri et al. [57], who have suggested that the high antibacterial efficacy of the immobilized Ag NPs is partly attributed to their small size, which enhances the faster dissolution and the more release of Ag^+^. Furthermore, immobilization allows for the contact-mode interaction of Ag NPs with a large number of bacterial cells, as the Ag NPs do not become sequestered inside the bacterial cells.

There was no difference between the growth inhibition of MRSA and *E*. *coli* by the composite in the agar diffusion assay (Figure 4C), which emphasizes two major aspects. First, the present composite exerts antibacterial effects against both Gram-positive and Gram-negative bacterial species tested, which is crucial in the context of wound dressing applications. Secondly, the composite shows antibacterial effects against the bacterial species most often involved in wound infections, especially the antibiotic-resistant bacterium, MRSA, posing a severe threat to wound management [14,33]. It has been suggested by Cutting et al. [32] that Ag dressings do not lead to a cure of infections, but rather they can efficiently inhibit bacterial penetration into wounds, due to their broad-spectrum of action. Accordingly, the present results suggest that the Ag–SiO_2_ composite can be used in wound dressing applications for the prophylaxis and control of antibiotic-resistant bacterial infections.

The most suitable decontamination method that retains the antibacterial effects of the composite was assessed by the parallel agar diffusion assays of the filter-sterilized Ag–SiO_2_ composite. Figure 4 shows that the UV-treated Ag–SiO_2_ composite produces larger IZs (11.5 ± 0.7 mm) of both strains tested than the filter-sterilized composite (8 ± 1.4 mm and 10 ± 1.4 mm against MRSA and *E*. *coli*, respectively). This is clearly due to the higher Ag concentration of the UV-treated composite compared to that of the filter-sterilized composite, as detected by ICP-OES. According to the present data, UV-irradiation is a robust method for decontaminating the composite and can be used to avoid the problem of clogging often observed when membrane filters are used.

**Figure 5:**
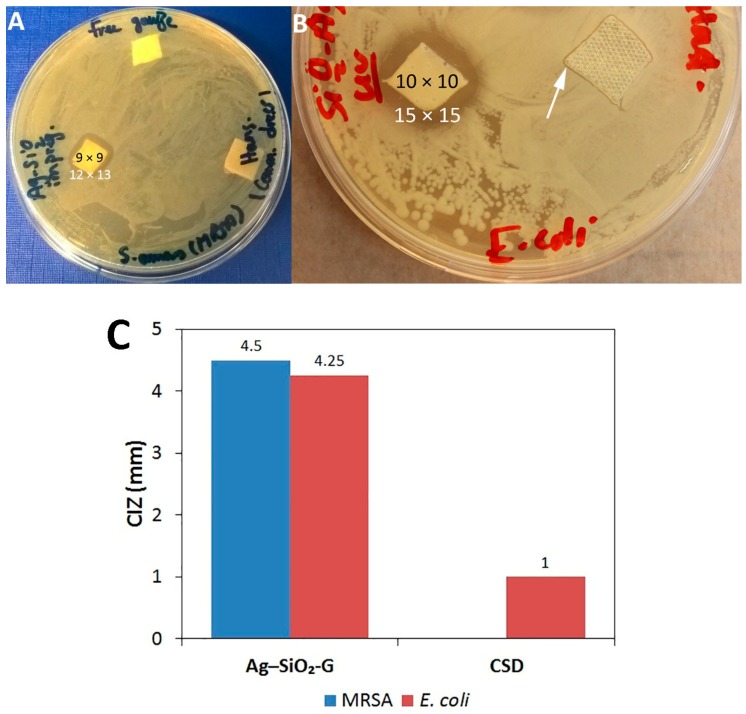
(**A**,**B**) Antibacterial effects of Ag–SiO_2_ composite-impregnated gauze (Ag–SiO_2_-G) against MRSA (**A**) and *E*. *coli* (**B**) in the agar diffusion assay. The black and white numbers represent the sizes of the dressings and the produced IZs in mm, respectively. The white arrow points to the small IZ produced by the commercial Ag-containing dressing (CSD) (Hansaplast) against *E*. *coli*. (**C**) The corrected inhibition zones (CIZs) of Ag–SiO_2_-G and CSD.

The efficacy of the composite in wound dressing applications was identified in the second set of agar diffusion assays. The Ag–SiO_2_-G is effective against both MRSA and *E*. *coli*, as clear IZs (Figure 5A,B, respectively) are observed after the gauze has been soaked in aqueous suspensions of the composite for only 15 min, highlighting the rapid and effective antibacterial action of the composite. Instead, no IZ is observed with the pristine control gauze (Figure 5A), indicating that the antibacterial effects of the Ag–SiO_2_-G are only attributed to the composite. The CSD was hydrated before testing to mimic the moist wound environment and was placed with its gray mesh side in contact with the inoculated plates to allow the release of Ag^+^ into the agar plates. However, the CSD does not inhibit the growth of MRSA (Figure 5A) and only slightly inhibits the growth of *E*. *coli* (Figure 5B); the produced IZ is far smaller than that produced by the Ag–SiO_2_-G. Figure 5C shows the remarkable differences between the corrected inhibition zones (CIZs) of the Ag–SiO_2_-G (4.5 and 4.25 mm against MRSA and *E*. *coli*, respectively) and CSD (1 mm only against *E*. *coli*). It has been suggested that hydration is required for an efficient leaching of Ag^+^ from Ag-containing dressings to achieve an antibacterial effect [44,52]. Liang et al. [58] have shown that Ag NPs on the hydrophilic surface of an asymmetric wettable AgNPs/chitosan composite dressing inhibit bacterial growth. In the present study, such favorable hydration conditions were maintained by soaking the gauze in an aqueous suspension of the composite with known concentration (1 mg/mL) for 15 min. However, the exact Ag concentration within the gauze after impregnation has not been determined. Moreover, the concentration of Ag within the CSD has not been elucidated. Therefore, further studies are necessary to determine the concentrations of Ag–SiO_2_ composites that are needed to impregnate the dressings in a manner that allows a sustained release and an effective antibacterial action of Ag. Collectively, the following aspects of our results are of importance: first, antibacterial effects are observed against both the Gram-positive MRSA and the Gram-negative *E*. *coli*. Secondly, the antimicrobial agar susceptibility test resembles the administration of dressings in the clinical settings and suggests that this bacterial growth inhibition can also occur at the wound-dressing interface. Thirdly, the hydration conditions that permitted the leaching of Ag^+^ can provide a basis for the use of Ag–SiO_2_-G as a topical wound dressing.

The minimum inhibitory concentrations (MICs) of the Ag–SiO_2_ composite against MRSA and *E*. *coli* are determined, as 250 and 500 μg/mL, respectively. Furthermore, the SiO_2_ particles show no inhibition of bacterial growth even at the highest concentration (1 mg/mL) tested, which further indicates that only the Ag in the Ag–SiO_2_ composite inhibits the bacterial growth. The present results advocate previous findings that SiO_2_ particles have no antibacterial effect [37,59]. The correlation between the MICs and the in vitro leaching profile of Ag from the composite shows promising antibacterial effects for the composite because a concentration of 1 mg/mL of Ag–SiO_2_ suspension has released 22.1 ± 2.4 μg/mL Ag after 24 h. It is evident that the MICs of 250 and 500 μg/mL of Ag–SiO_2_ composite have released ~5.5 and 11.1 μg/mL Ag, respectively, after overnight incubation in the broth microdilution test. Hence, it can be argued that ~5.5 and 11.1 μg/mL are the elemental Ag concentrations that should be leached from the Ag–SiO_2_ composite at their prolonged antibacterial administration in wound dressings to inhibit the growth of MRSA and *E*. *coli*, respectively. The present MICs are encouraging, as based on their elemental Ag concentrations, they are less than the previously reported MICs of Ag–SiO_2_ composites with pure Ag concentration of 50 and 12.5 μg/mL against *S*. *aureus* and *E*. *coli*, respectively [43], and 6.72 to 13.44 μg/mL against *S*. *aureus* [27]. Contrary to expectations that Gram-positive bacteria are less susceptible to Ag–SiO_2_ composites than Gram-negative bacteria [35,41,42], owing to the thicker cell wall of Gram-positive bacteria [27,43]. The present study did not find remarkable differences in the susceptibility of MRSA and *E*. *coli* to the composite in the agar diffusion assays. Moreover, based on the MICs, the Gram-positive MRSA is even more susceptible to the composite than the Gram-negative *E*. *coli*. All of the results described so far in the present study indicate that the Ag–SiO_2_ composite displays eminent antibacterial effects against representatives of both Gram-positive and Gram-negative bacteria. Dong et al. [7] have demonstrated that Ag–SiO_2_/poly-ε-caprolactone nanofibrous membranes promote good and fast wound healing, with less inflammation and epithelial shrinkage of wounds induced in Wistar rats, which was attributed to the antibacterial effects of the released Ag–SiO_2_. The aforementioned study utilized a previously synthesized Ag–SiO_2_ composite with small-sized Ag NPs (2 to 10 nm) and a MIC of 6.72 to 13.44 μg/mL elemental Ag against *S*. *aureus* [27]. The composite synthesized in our study is composed of small-sized Ag NPs (5 nm) and has a low MIC of ~5.5 μg/mL elemental Ag against MRSA. In light of the findings of Dong et al. [7], a role for the Ag–SiO_2_ composite in wound healing in vivo seems plausible and further studies are warranted.

**Figure 6:**
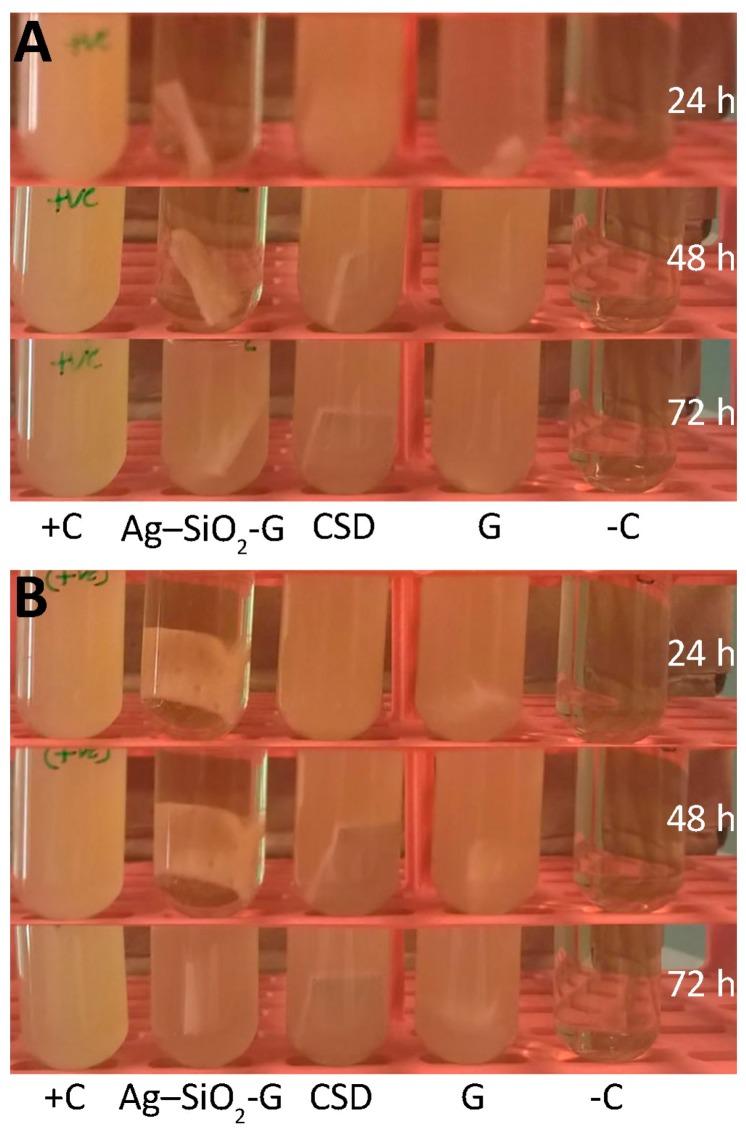
The prolonged antibacterial effects of Ag–SiO_2_-G against (**A**) MRSA and (**B**) *E*. *coli* in the Mueller–Hinton broth (MHB) turbidity assays observed after 24, 48, and 72 h of incubation. G, pristine gauze. +C, bacterial suspensions without dressings. –C, MHB without bacteria.

**Figure 7:**
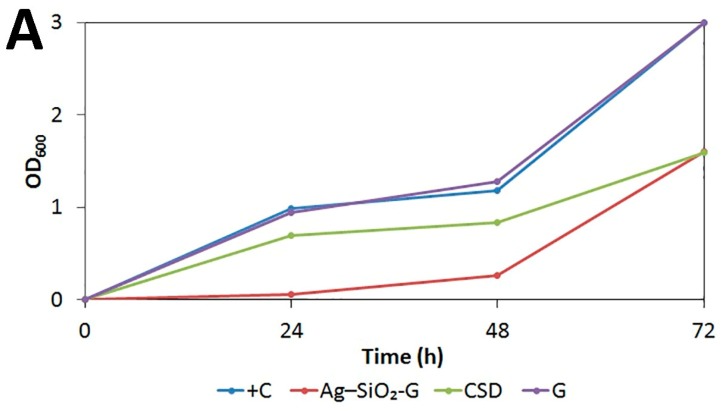

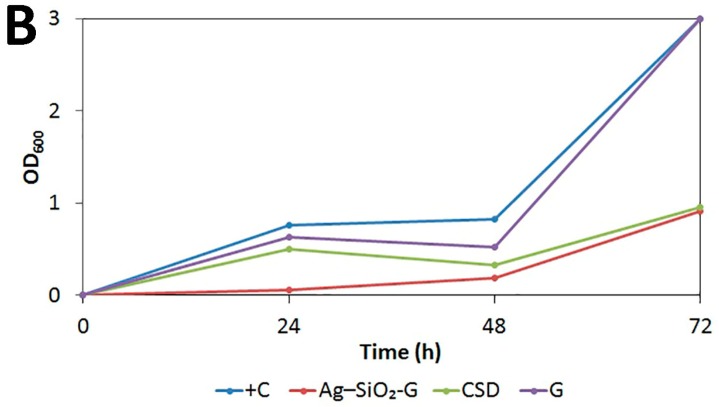
Growth curves of (**A**) MRSA and (**B**) *E*. *coli* in MHB in the presence of no inhibitor (+C), Ag–SiO_2_-G, CSD, and pristine gauze (G). Each data point represents the average of five consecutive measurements. The standard errors were too small to be depicted. The data shown is a representative of two independent experiments.

The prolonged antibacterial effects of Ag–SiO_2_-G are shown in the turbidity assays (Figure 6); the quantitative results are shown in Figure 7 and Table S1. When comparing the growth of the bacterial cultures containing different dressings to that of the positive controls, it is clear that the Ag–SiO_2_-G inhibits the growth of MRSA and *E*. *coli* after 24 h, and powerfully reduces their proliferation after 48 h, indicating prolonged antibacterial effects of the Ag–SiO_2_-G. This prolonged antibacterial effect is required in practical applications [35] and desired feature in Ag-containing dressings [44], decreasing the frequency of dressing changes [60]. The CSD only slightly delayed the bacterial growth after 24 and 48 h, indicating a far less lasting antibacterial effect when compared to the Ag–SiO_2_-G. The lack of any antibacterial effect of the pristine gauze was further confirmed by that the bacterial cultures containing pristine gauze reached almost the same turbidity and bacterial growth as the positive controls. The present findings point to the prolonged antibacterial effects of the Ag–SiO_2_-G against both the Gram-positive MRSA and Gram-negative *E*. *coli* that are promising for wound dressing applications.

**Figure 8:**
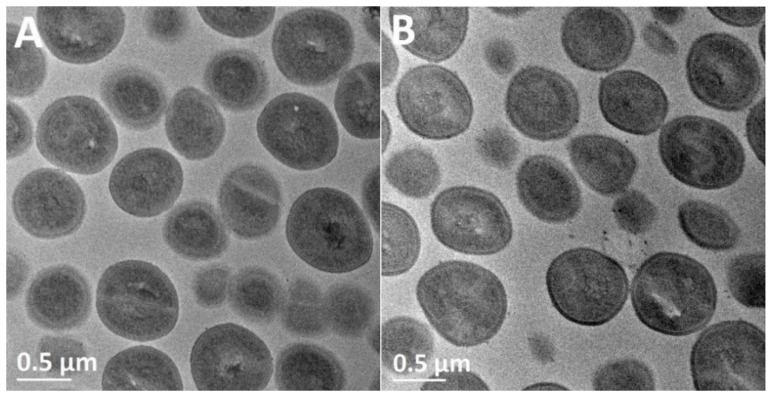

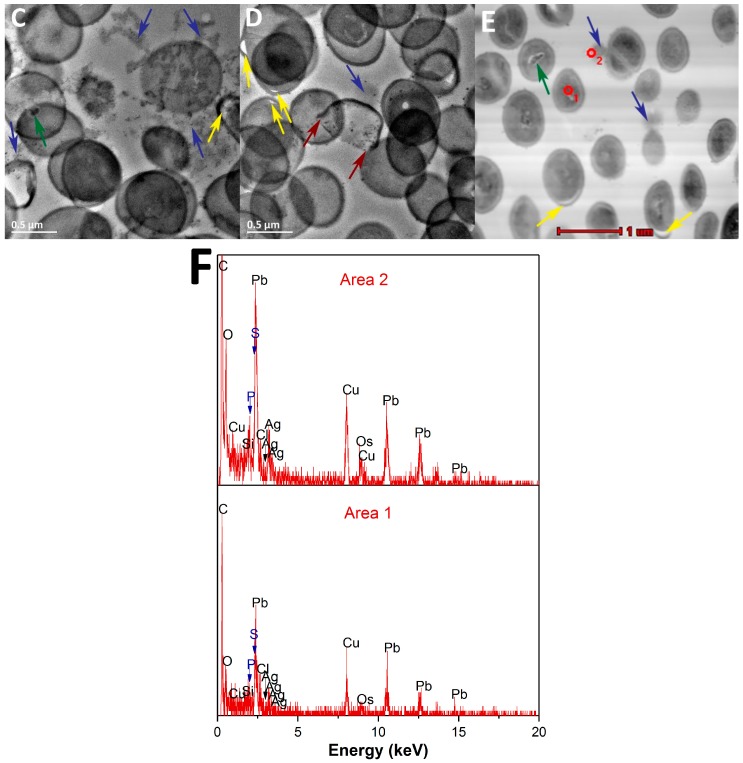
TEM images of (**A**) untreated MRSA; (**B**) MRSA treated with pristine SiO_2_ NPs; and (**C**,**D**) MRSA treated with the Ag–SiO_2_ composite; (**E**) HAADF-STEM image of MRSA treated with the composite; (**F**) The EDX elemental analyses of the selected areas 1 and 2 in panel E. Yellow arrows highlight the gaps between the cell walls and cytoplasmic membranes. Blue arrows show the release of cytoplasmic contents from the bacterial cells. Green arrows demonstrate the central condensation of the bacterial DNA. Red arrows indicate the disruption and loss of bacterial membranes.

The mechanisms of the antibacterial effects of the composite are elucidated in Figure 8 using TEM and STEM. Figure 8A shows the normal coccal morphological structure of the untreated MRSA with the intact cell walls and cytoplasmic membranes. Figure 8B presents the same normal morphological structures of MRSA after treatment with pristine SiO_2_ particles, relating the antibacterial effects of the composite to the Ag NPs at the microscopic level as well. In contrast, MRSA treated with the Ag–SiO_2_ composite (Figure 8C,D) furnished the scenery with a series of morphological changes, including (i) gaps between the bacterial cell walls and cytoplasmic membranes; (ii) the release of cytoplasmic contents from the bacterial cells; (iii) the disruption and loss of bacterial membranes; and (iv) the central condensation of the bacterial DNA. Some morphological changes are similarly shown in the high-angle annular dark-field scanning transmission electron microscope (HAADF-STEM) image (Figure 8E) with inverse contrast. STEM with EDX is a sophisticated analytical tool allows for studying the elemental composition of the composite-treated MRSA. Figure 8F demonstrate the EDX qualitative chemical analyses, corresponding to the interior and the released cytoplasmic contents of composite-treated MRSA, respectively. The EDX spectra show that Ag was detected in both areas selected, together with phosphorus (P) and sulfur (S). Si and O peaks originate from the SiO_2_ matrix. Carbon (C) and Cu peaks originate from the grid. Chlorine (Cl) peaks are artifacts from the preparation of the sample. Osmium (Os) peaks arise from osmium tetroxide used for the fixation of bacterial cells. Lead (Pb) peaks arise from lead citrate used for staining of the bacterial cells.

To date, studies investigating the exact mechanism of antibacterial effects of Ag NPs have produced equivocal results. The antibacterial effects of Ag NPs could be attributed to: (i) the Ag NPs themselves in the immobilized or colloidal forms; or (ii) the released Ag^+^ from the Ag NPs [57]. A link has been drawn between the positive charge of the Ag–SiO_2_ NPs and the produced antibacterial effects against *S*. *aureus* and *E*. *coli* [27] as positively charged surfaces exhibit an electrostatic attraction to the negatively charged bacterial cells, allowing initial bacterial adhesion [61]. This is, however, not consistent with our findings, as the Ag–SiO_2_ composite used in the present study is negatively charged. Prior studies have also noted the damage of bacterial membranes at treatment with Ag NPs as “pits” and gaps were formed in the cell wall peptidoglycan of *S*. *aureus* [62] and in the outer membranes of *E*. *coli* [28,63]. In contrast to earlier findings, in the present study, no evidence of “pit” formation is detected. However, the cytoplasm is released from the bacterial cells without the destruction of the bacterial membranes (Figure 8C), and finally, the loss of the bacterial membranes (Figure 8D) is detected. The present results can be due to the antibacterial effects of the released Ag^+^ from the Ag NPs of the composite, interacting inside the bacterial cells. Ag^+^ can permeate into the bacterial cells through the ion channels without destructing the bacterial membranes [64]. On the other hand, the observed central condensation of the bacterial DNA (Figure 8C,E) and the presence of P and S in the EDX spectra (Figure 8F) further support the ideas of Feng et al. [30], who have also detected P and S in *S*. *aureus* treated with Ag^+^. They have suggested that Ag^+^ causes (i) the condensation of DNA (constituted of a high amount of P), leading to the loss of replication ability; and (ii) an interaction between Ag^+^ and thiol groups of bacterial proteins, resulting in protein inactivation and bacterial cell wall damage, or even complete cell wall loss at the final stage. Taken together, the present findings have important implications for the understanding of how the Ag–SiO_2_ composite exerts its antibacterial effects. Namely, the released Ag^+^ interact with the bacterial cytoplasmic constituents, leading ultimately to the disruption and loss of bacterial membranes.

## 3. Materials and Methods 

### 3.1. Materials

Tetraethyl orthosilicate (TEOS, ≥99.0%) and silver nitrate (≥99.0%) were obtained from Sigma-Aldrich (Steinheim, Germany and St. Louis, MO, USA, respectively). Ammonium hydroxide (25%) and ethanol (EtOH, 96.1 vol %) were purchased from JT Baker (Phillipsburg, NJ, USA) and Altia (Rajamäki, Finland), respectively. Cellulose acetate membranes (25 mm syringe filter *w*/0.2) were obtained from VWR International (Wallkill, NY, USA). *Staphylococcus aureus* subsp. *aureus* (MRSA, ATCC 43300, KWIK-STIK) was purchased from Microbiologics (St. Cloud, MN, USA), and *E*. *coli* (VTT E-94564) was provided by the culture collection of the Department of Bioproducts and Biosystems, School of Chemical Engineering, Aalto University. Luria–Bertani (LB) broth and LB agar were purchased from BD Difco (Franklin Lakes, NJ, USA). Mueller–Hinton broth (MHB) and Mueller–Hinton agar (MHA) were purchased from Lab M Limited (Heywood, Lancashire UK). The pristine gauze (Mepore) and the CSD (Hansaplast, Sensitive MED XXL Antibacterial Plaster) were manufactured by Mölnlycke Health Care (Gothenburg, Sweden) and Beiersdorf AG (Hamburg, Germany), respectively. According to the manufacturer, the Hansaplast MED plasters are non-adhesive wound pads, containing Ag-coated polyethylene nets releasing Ag^+^ at contact with the wound fluid.

### 3.2. Preparation of Ag–SiO_2_ Composite and SiO_2_ Particles

The preparation of the SiO_2_ particles was performed by the Stöber method [65]. The Ag–SiO_2_ composite was prepared using the previously reported procedure [66]. In brief, 1000 mL EtOH, 100 mL deionized water, and 100 mL ammonium hydroxide were mixed in a large beaker. Then, 2 g of silver nitrate was dissolved in the aforementioned solution, followed by the addition of 50 mL TEOS, which turned the solution white. The SiO_2_ particles were prepared using the same aforementioned procedure without the addition of silver nitrate. Both solutions were left to react for 2 h and centrifuged at 3500 rpm. The prepared powders were dried at room temperature and heat-treated at 300 °C for 75 min.

### 3.3. Characterization of Ag–SiO_2_ Composite and SiO_2_ Particles

The structures of the Ag–SiO_2_ composite and SiO_2_ particles were studied by XRD using a PANalytical X’pert Powder Pro diffractometer with Cu Kα radiation (λ = 1.54 Å) over the 2*θ* range of 20° to 90°. The surface morphology of the Ag–SiO_2_ composite and that of the SiO_2_ particles were examined using a field-emission gun scanning electron microscope (FEG-SEM, Hitachi S-4700, Tokyo, Japan). The shape and distribution of Ag NPs on the SiO_2_ matrix was detected by a TEM (Tecnai F20 G2, Eindhoven, The Netherlands) operated at 200 kV accelerating voltage. The crystal structure of the Ag NPs on the composite was investigated by the electron diffraction ring pattern and the morphology was examined by HRTEM. The chemical structure of the composite was qualitatively examined by the EDX unit of the STEM. The size distributions of the pristine SiO_2_ particles and the Ag NPs of the composite were analyzed using the obtained SEM and TEM images, respectively, by ImageJ software (National Institutes of Health, Bethesda, MD, USA). The zeta (ζ) potentials of the Ag–SiO_2_ composite and SiO_2_ particles dispersed in Milli-Q water were analyzed by a Zetasizer Nano ZS (Malvern, UK); the results were based on the average of five measurements.

### 3.4. Ag Leaching from Ag–SiO_2_ Composite

ICP-OES (PerkinElmer Optima 7100 DV, Waltham, MA, USA) was utilized to measure the Ag concentrations leached from the Ag–SiO_2_ composite over three successive days. First, the total Ag concentration in 1 mg/mL aqueous suspension of the Ag–SiO_2_ composite (non-filtered stock) was determined after dissolving the Ag of the composite in equal volumes of 65% nitric acid (HNO_3_). Secondly, the prolonged leaching was detected as follows: aqueous suspensions of the Ag–SiO_2_ composite (1 mg/mL) were shaken at 150 rpm (Lab-Therm, Fennolab, Kühner, Switzerland) for 0, 24, 48, and 72 h. After which, the shaken suspensions were filtered through 0.2 μm cellulose acetate membranes to remove the SiO_2_ particles and the concentrations were measured. The measurements were conducted in triplicate.

### 3.5. Antibacterial Tests

MRSA and *E*. *coli* were cultured overnight at 37 °C on LB agar. Disinfection of the Ag–SiO_2_ composite and SiO_2_ powders was performed by UV-irradiation at room temperature for 12 h (Biowizard Silver Line, Kojair, Vilppula, Finland). Then, all the UV-treated powders were dispersed in sterile Milli-Q water at the concentration of 1 mg/mL. The dispersed materials were sonicated for 30 min (Bransonic, 2210E-DTH, Danbury, CT, USA, power 234 W, working frequency 47 kHz ± 6%) before the antibacterial tests to obtain homogeneous solutions. The antibacterial tests were performed under aerobic conditions. To obtain information about the most suitable decontamination method for the composite, the Ag–SiO_2_ composite was also sterilized by filtration through a 0.2 μm cellulose acetate membrane.

#### 3.5.1. Agar Diffusion Assays 

The antimicrobial agar susceptibility tests were performed according to the recommendations of the Clinical and Laboratory Standards Institute (CLSI) [67]. An aliquot of 100 μL of each bacterial suspension of ~1 to 2 × 10^8^ colony-forming units (CFU)/mL was spread on the MHA plates. Then, 100 μL of the Ag–SiO_2_ and SiO_2_ solutions tested were dispensed into the 5 mm-diameter wells of the plates. The agar diffusion assays were performed in duplicate and parallel agar diffusion assays were performed for the filter-sterilized Ag–SiO_2_ composite. The diameters of IZs (mm) were measured after overnight incubation at 37 °C. 

To establish the potential of the composite for practical wound dressing applications, the antibacterial effects of the Ag–SiO_2_ composite-impregnated gauze (Ag–SiO_2_-G) were experimented in another set of antimicrobial susceptibility tests and compared with the commercial Ag-containing dressing (CSD, Hansaplast). The sterile gauze (Mepore) was cut under aseptic conditions into quadrate pieces of approximately 1 cm × 1 cm. Each piece was soaked in a sterile vial containing 1 mg/mL of the Ag–SiO_2_ composite for 15 min. Quadrate pieces (1 cm × 1 cm) of the CSD and, as a control, the pristine gauze (Mepore not impregnated with the Ag–SiO_2_ composite) were soaked in vials containing only sterile Milli-Q water. The pieces of the Ag–SiO_2_-G were placed on the surface of the inoculated MHA plates to detect the inhibition of bacterial growth. The gray mesh sides of the CSD pieces were placed in contact with the inoculated surfaces of the plates. The inhibition of bacterial growth was detected after overnight incubation. The agar diffusion assays for the wound dressings were performed in duplicate. For this set of experiments, CIZs [52,68] were calculated to take into account both horizontal and vertical IZs and to control the error originating from cutting the pieces. The calculation was executed as follows: (i) the IZs (mm) were measured horizontally and vertically and calculated as the average of measurements; (ii) the average size of dressings was similarly measured; (iii) the CIZs were calculated by subtracting the average size of dressings from the average of IZs.

#### 3.5.2. Broth Microdilution Method 

The standard broth microdilution method was utilized to determine the MICs of the Ag–SiO_2_ composite and SiO_2_ particles according to the recommendations of the CLSI [69]. The composite and SiO_2_ particles were twofold serially diluted from 1 mg/mL to 31.25 μg/mL in MHB. An aliquot of 100 μL of each concentration of the different materials tested was added into the wells of the microtiter plate. Then, 10 μL of the bacterial suspensions (5 × 10^6^ CFU/mL) were inoculated into the wells to reach the final bacterial concentration of 5 × 10^5^ CFU/mL in each well of the microtiter plate. Pure MHB was utilized as a negative control and bacterial suspensions without any additions were utilized as positive controls. The MICs were recorded after overnight incubation at 37 °C.

#### 3.5.3. Prolonged Antibacterial Effects of Ag–SiO_2_-G

The antibacterial effects of the Ag–SiO_2_-G were assessed over three successive days by a modified method from a previously reported procedure [70]. Briefly, quadrate pieces (1 cm × 1 cm) of the prepared Ag–SiO_2_-G, CSD, and the pristine gauze were pretreated in sterile test tubes containing 800 μL of sterile de-ionized water for 10 min and then 2.2 mL of MHB was added to each test tube yielding a total volume of 3 mL. An aliquot of 10 μL of MHB-bacterial suspensions (~1 to 2 × 10^8^ CFU/mL) was added to the test tubes containing the dressings. The test tubes were incubated at 37 °C with shaking (200 rpm). The test tube containing MHB without cultured bacteria was utilized as a negative control. The test tubes containing bacterial suspensions in MHB without dressings were utilized as positive controls. The prolonged antibacterial effects were observed every 24 h of incubation by (i) the visual inspection of the test tubes for turbidity and (ii) the quantitative measurements of bacterial growth kinetics at the optical density (OD) of 600 nm, in reference to the negative control and positive controls. The OD was calculated as the average of five measurements.

#### 3.5.4. Mechanisms of Antibacterial Effects of Ag–SiO_2_ Composite

In order to identify the possible mechanisms of antibacterial effects of the composite, MRSA was treated with the Ag–SiO_2_ composite and the pristine SiO_2_ particles for morphological observations using TEM and X-ray microanalyses using STEM. Untreated MRSA was utilized as a negative control. MRSA was cultured in LB broth (~1 to 2 × 10^8^ CFU/mL) with shaking (200 rpm) at 37 °C overnight. Aliquots of 500 μL of the Ag–SiO_2_ and SiO_2_ solutions were added to the bacterial suspensions, and the incubation was continued for 24 h. The bacterial cells were centrifuged and washed, and further processed by fixation (first in 2.5% glutaraldehyde in 0.1 M sodium cacodylate buffer at 4 °C for 24 h, then with 1% osmium tetroxide at room temperature for 1 h), dehydration, infiltration, and polymerization, as previously reported [59]. Following polymerization, the epon blocks were cut into 60 nm thick sections, using a Leica ultramicrotome (EM Ultra Cut UC6ei, Leica Mikrosysteme GmbH, Vienna, Austria). The sections were drop-cast on grids (formvar-coated 200-mesh EM copper grids, Electron Microscopy Sciences, Hatfield, PA, USA) and first stained with 0.5% uranyl acetate, then with 3% lead citrate.

## 4. Conclusions

We have immobilized Ag NPs, with small sizes and uniform distribution, on a SiO_2_ matrix and characterized the developed Ag–SiO_2_ composite by a variety of instrumental techniques. The composite displayed a rapid Ag leaching after 24 h followed by a slower prolonged leaching. The evaluation of the antibacterial effects of the composite resulted in the following key findings: (i) the composite has antibacterial effects against both MRSA and *E*. *coli*; (ii) the MICs of the composite indicate eminent antibacterial effects with reference to the released Ag concentrations; (iii) the Ag–SiO_2_-G has antibacterial effects superior to those of a CSD; (iv) the Ag–SiO_2_-G has prolonged 48 h antibacterial effects, important for wound dressing applications; and (v) the composite exerts its antibacterial effects through the released Ag^+^ interacting with the phosphorus of DNA, losing its replication ability, and the thiol groups of proteins, causing ultimately the loss of bacterial membranes. These data suggest a major potential for the use of the Ag–SiO_2_ composite in the development of wound dressings for acute wound management and infection control. The findings of the present study have directed our interest, as a natural progression of this work, to further investigating the possible cytotoxic effects of the composite on skin cells. We will also impregnate the composite into cellulose membrane dressings, and investigate the in vivo wound healing capacity of the composite-impregnated membranes in animal models.

## Figures and Tables

**Table 1:** The sizes of SiO_2_ particles and Ag NPs on the composite. The number of measured particles is 50 at a minimum for each sample. standard deviation (SD).

Materials	SiO_2_ Particles	Ag Nanoparticles (NPs) of the Composite
Median size (nm)	673	5
Mean size (nm)	674	5
SD (nm)	22	2
Minimum particle size (nm)	616	2
Maximum particle size (nm)	724	20

## Supplementary Materials

The following are available online at http://www.mdpi.com/2079-4991/7/9/261/s1, Figure S1: X-ray diffraction (XRD) analyses of the nanosilver–silica (Ag–SiO_2_) composite and silica (SiO_2_) particles. Figure S2: Size distribution of the Ag NPs of the Ag–SiO_2_ Composite. Figure S3: High-resolution transmission electron microscope (HRTEM) image of the Ag–SiO_2_ composite. Table S1. Prolonged antibacterial effects of Ag–SiO_2_-G.
