# Supplementary material for: Nanosilver–Silica Composite: Prolonged Antibacterial Effects and Bacterial Interaction Mechanisms for Wound Dressings
Source: Nanomaterials (Basel). 2017 Sep 6;7(9):261. doi: 10.3390/nano7090261 (PMC5618372; doi:10.3390/nano7090261)
Supplement: Supplementary file 1 [file nanomaterials-07-00261-s001.pdf]

Supplementary Material

Nanosilver–Silica Composite: Prolonged Antibacterial Effects and Bacterial Interaction Mechanisms for Wound Dressings

Dina A. Mosselhy[1,2,*], Henrika Granbohm[1], Ulla Hynönen[3], Yanling Ge[1], Airi Palva[3], Katrina Nordström[4] and Simo-Pekka Hannula[1]

[1] Department of Chemistry and Materials Science, School of Chemical Engineering, Aalto University, 02150 Espoo, Finland
[2] Microbiological Unit, Fish Diseases Department, Animal Health Research Institute, Dokki, 12618 Giza, Egypt
[3] Department of Veterinary Biosciences, Division of Veterinary Microbiology and Epidemiology, University of Helsinki, P.O. Box 66, 00014 Helsinki, Finland
[4] Department of Bioproducts and Biosystems, School of Chemical Engineering, Aalto University, 02150 Espoo, Finland
* Correspondence: dina.mosselhy@aalto.fi; Tel.: +358-50-408-3533

X-ray diffraction (XRD) analyses of the nanosilver-silica (Ag–SiO_2) composite and silica (SiO_2) particles:

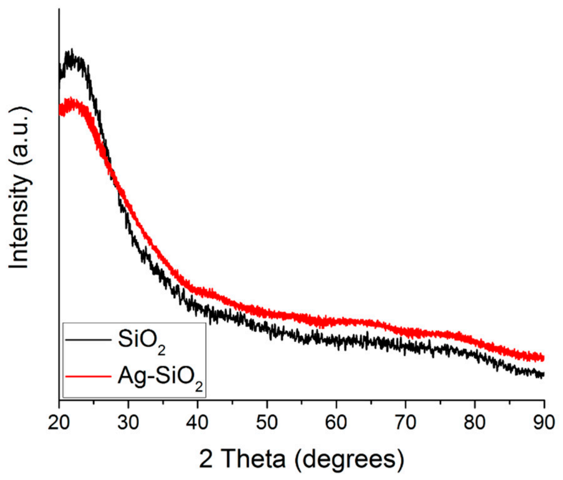

Figure S1. XRD patterns of the Ag–SiO_2 composite (heat-treated at 300 °C for 75 min) and SiO_2 particles.

Size distribution of the Ag NPs of the Ag–SiO_2 Composite

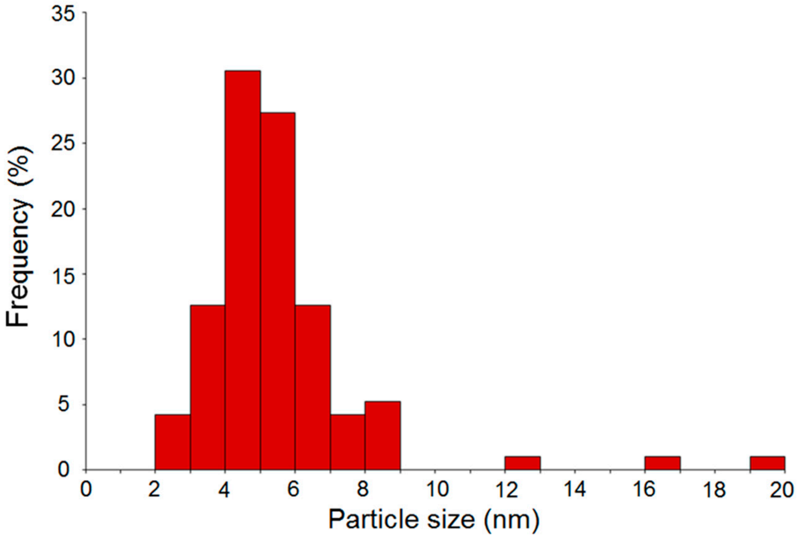

Figure S2. Size distribution histogram of the Ag NPs of the Ag–SiO_2 composite. The median size of the Ag NPs is 5 nm based on the analysis of 95 Ag NPs on multiple TEM images.

High-resolution transmission electron microscope (HRTEM) image of the Ag–SiO_2 composite

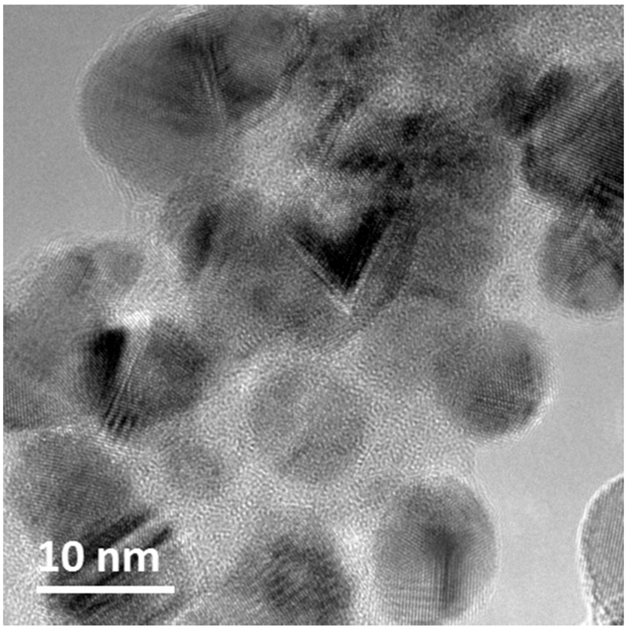

Figure S3. HRTEM image of the Ag–SiO_2 composite showing twinned and multi-grain Ag NPs.

Table S1. Prolonged antibacterial effects of Ag–SiO_2-G.

Inhibitor in MHB	OD at 600 nm (average ± SD)					
	24 h		48 h		72 h	
	MRSA	*E. coli*	MRSA	*E. coli*	MRSA	*E. coli*
**None**	0.981 ± 0.001	0.756 ± 0.001	1.18 ± 0.002	0.822 ± 0.002	3	3
**Ag–SiO_2-G[1]**	0.053 ± 0.001	0.051 ± 0.001	0.256 ± 0.001	0.182	1.604 ± 0.013	0.913 ± 0.002
**CSD[2]**	0.688 ± 0.002	0.496 ± 0.001	0.837 ± 0.002	0.33 ± 0.002	1.588 ± 0.016	0.957 ± 0.003
**G[3]**	0.938 ± 0.002	0.629 ± 0.002	1.282 ± 0.007	0.518 ± 0.002	3	3

[1] Ag–SiO_2 composite-impregnated gauze. [2] Commercial Ag-containing dressing. [3] Pristine gauze.
